# Short report: Targeted analysis of whole exome sequencing data in Indian cryptogenic stroke patients

**DOI:** 10.1371/journal.pone.0326554

**Published:** 2026-02-20

**Authors:** Priya Dev, Jenefer M. Blackwell, Rajiv Kumar, Vijay Mishra, Abhishek Pathak

**Affiliations:** 1 Department of Neurology, Institute of Medical Sciences, Banaras Hindu University, Varanasi, India; 2 The Kids Research Institute Australia, University of Western Australia, Perth, Western Australia, Australia; 3 Centre of Experimental Medicine & Surgery, Institute of Medical Sciences, Banaras Hindu University, Varanasi, India; Mount Sinai Medical Center, UNITED STATES OF AMERICA

## Abstract

Cryptogenic stroke (CS) is an ischemic stroke of unknown cause with increasing incidence in India. Common and rare genetic variants have been associated with the risk of stroke. We carried out targeted analysis of whole exome sequencing on a small cohort of 16 CS patients compared to 16 healthy unaffected relatives to determine whether rare coding variants in genes previously associated with stroke could play a role in India. Variants were filtered for coverage (≥20x) and minor allele frequency (≤0.01). Putative deleterious variants were identified using a range of bioinformatic tools. Targeted analysis was performed by filtering for those variants present in a panel of 220 stroke-related genes. Phenotypes, pathways and cell compartments to which genes carrying putative deleterious (PHRED-scaled CADD scores ≥15) variants belonged were determined using Enrichr. STRING was employed to identify interacting proteins. We identified 17 potentially damaging variants specific to Indian CS patients in 15 genes contributing to phenotypes (e.g., hemorrhage; abnormal blood coagulation; dilated aorta, increased heart weight) and pathways (e.g., platelet degranulation, common pathway of fibrin clot formation; response to elevated platelet cytosolic Ca^2+^) that were not observed in unaffected relatives. STRING analysis identified 6 genes (*ITGA2B*, *F13A1*, *F5, ATP7A, GLA, ABCC6*) encoding interacting proteins that could be prioritised for follow-up studies. This should include secondary sequence validation, as well as extended pedigree and functional laboratory-based gene-editing studies to validate the clinical relevance of specific variants to CS. Although limited by small sample size, our study provides novel data on CS in a geographical region and ethnic group not well studied to date.

## Introduction

Stroke is the second largest cause of death and the third largest cause of years of life lost worldwide. Ischemic stroke, an overt symptomatic expression of brain infarction, accounts for ~80% of all strokes, with most cases caused by a combination of environmental and genetic factors. Cryptogenic (unexplained) stroke (CS) accounts for ~30%–40% of ischemic stroke patients, and is increasing in the Indian population [1]. A better definition and identification of associated risk factors are required to manage CS.

Heritability for ischemic stroke is substantial (37.9%) and varies with subtype (40.3% for large-vessel disease; 32.6% for cardioembolic; and 16.1% for small vessel disease) [2]. Genome-wide studies have identified common variants in a large number of genes associated with stroke, including in India [3]. Rare genetic variants in monogenic disorders can also lead to ischemic stroke (OMIM#601367). Evidence is accumulating for the contribution of rare functional coding variants to genetic risk in complex diseases [4], while next-generation sequencing has made finding these more cost-effective. Here we employ targeted analysis of whole exome sequencing (WES) data in Indian CS patients to determine whether rare putative deleterious variants in previously identified stroke genes could play a role in disease risk.

## Materials and methods

All procedures in this study were conducted according to the principles of the Declaration of Helsinki. This study was approved by the Institutional Ethics Committee, Institute of Medical Sciences (IMS), Banarus Hindu University (BHU), Varanasi, India with reference number: Dean/2018/EC/288. Patients were enrolled between 26 August 2020 and 30 March 2022. Written consent for participation was obtained from all the patients/persons responsible. All persons whose DNA was collected for this research consented to storage of the sample and future use of de-identified genetic and clinical data. All participants agreed to publication of de-identified genetic and clinical data.

### Study subjects

The study was conducted at the Department of Neurology, IMS, Sir Sunderlal Hospital, BHU, Varanasi, India. We enrolled 16 consecutive CS patients (age range 47–84 years; 10 males, 6 females) with ischemic strokes of undetermined etiology. Samples were also collected from 16 unaffected family members (age range 20–59 years; 14 males, 2 females) for comparative analyses. This was not an extended family study *per se*, but data from unaffected family members (one per patient) were used to identify variants specific to CS patients.

CS was defined as an ischemic stroke not attributed to a definite source of large-vessel atherosclerosis, cardioembolism, or small vessel disease, according to the Trial of ORG 10172 in Acute Stroke Treatment (TOAST) classification [5]. Patients presenting within 48h of onset of clinical symptoms and with a modified Rankin Scale (mRS) score ≥1 at admission were included. Complete evaluation, including clinical assessment and neurological examination, was carried out in line with the National Institutes of Health Stroke Scale within 60 min of the patient’s arrival. The diagnostic assessment included non-contrast computed tomography or brain magnetic resonance imaging, hemogram, biochemical tests, electrocardiogram, transoesophageal echocardiography, vascular imaging (of intra- and extra-cranial vessels), assessment of prothrombotic state, and 24h Holter monitoring for atrial fibrillation. Patients with conditions requiring intensive care unit management, pregnancy, recurrent ischemic stroke, subarachnoid hemorrhage, traumatic brain injury, vascular aneurysm, arterial malformation, infective endocarditis, central nervous system infections or chronic liver or kidney diseases were excluded.

### Library preparation and exome sequencing

DNA was extracted from 2 ml of peripheral blood using Qiagen DNA mini kits, with quantity/quality of genomic DNA (gDNA) measured by NanoDrop-2000 Spectrophotometer. DNA samples were sent to Dr Lal PathLabs Ltd (New Delhi, India) where library construction, sequencing and data analyses were undertaken. Libraries were constructed from 100ng gDNA using the Ion Ampliseq Exome RDY Panel kit (Thermo Fisher Scientific) and quantified with Qubit™ dsDNA HS (High Sensitivity) Assay Kit on Qubit 3.0 Fluorometer. Templates were generated using 25 pm of each library (Ion Chef Instrument; Thermo Fisher Scientific), followed by enrichment of templated ion sphere particles. WES was performed using Hi-Q chemistry on the Ion Proton system (Thermo Fisher Scientific).

### Data processing and variant analysis

Sequences were aligned against the reference genome (GRCh37/hg19) using Torrent Suite v.5.12.0 and Variant Caller v.5.2.1 software, including coverage analysis and variant caller plugins (Thermo Fisher Scientific). The average (± standard deviation) coverage for CS patients was 93.49 ± 1.97%, for unaffected relatives 93.06 ± 1.71%. Variant discovery, genotype calling of multi-allelic substitutions and indels were performed using the Torrent Variant Caller version 4.6.0.7. The Torrent Coverage Analysis provided statistics and graphs describing the level of sequence coverage produced for targeted genomic regions (version 4.6.0.3). The Annotate variants 5.0 of Ion Reporter (Thermo Fisher) annotated the variants. The average (± standard deviation) total exonic variants for CS patients was 20749 ± 2562 (9840 ± 1470 nonsynonymous SNVs; 10547 ± 1024 synonymous SNVs; 361 ± 73 insertion/deletions) and for unaffected family members was 19880 ± 2058 (9340 ± 1122 nonsynonymous SNVs; 10209 ± 890 synonymous SNVs; 331 ± 58 insertion/deletions).

### Variant prioritization and bioinformatics analysis

An Integrative Genome Viewer (https://www.broadinstitute.org/igv/) was used to visualise sequencing data. Variant frequencies were obtained from public domain databases including the 1000 Genomes Project (https://www.internationalgenome.org/) and the Genome Aggregation Database (gnomAD; https://gnomad.broadinstitute.org/). Variants detected in the exome sequencing were filtered for coverage (≥20x) and minor allele frequency (≤0.01) in the public domain databases. All were compared with mutation databases including the Human Gene Mutation Database; https://www.hgmd.cf.ac.uk/ac/index.php/) and Uniprot (https://www.uniprot.org/). Intronic, up/downstream, and synonymous variants were removed. Predicted deleteriousness of the detected variants was evaluated using DEOGEN2 (https://deogen2.mutaframe.com/), Mutation Taster (https://www.mutationtaster.org/), Sorting Intolerant From Tolerant (SIFT; https://sift.bii.a-star.edu.sg/), Protein Variant Effect Analyzer (https://provean.jcvi.org/index.php), Functional Analysis Through Hidden Markov Model (https://fathmm.biocompute.org.uk/) and Deleterious Annotation of Genetic Variation using Neural Networks (Index of/public_data/DANN). Variants predicted to be deleterious, i.e., damaging, disease-causing, or likely pathogenic using these tools, were taken forward in the targeted analysis.

### Targeted exome analysis

For targeted analysis variants predicted to be deleterious were filtered against an in-house gene panel (Dr Lal Pathlabs Ltd) of 220 genes (S1 Table) previously identified as risk factors for stroke and related clinical phenotypes. The average (± standard deviation) quality threshold for the coverage across the targeted region for CS patients was 97.62 ± 1.73%, for unaffected relatives 97.56 ± 1.77%, with read depths > 20x. All filtered variants in these genes were separately examined in ClinVar (https://www.ncbi.nlm.nih.gov/clinvar/), the public archive of interpretations of clinically relevant variants, which classified the variants identified as pathogenic or variant of uncertain signficiance (VUS), according to American College of Medical Genetics and Genomics (ACMG) 2015 guidelines. As a measure of putative deleteriousness that correlates well with the qualitative bioinformatic assessment of deleteriousness provided by the suite of tools employed by the sequencing company we report PHRED-scaled Combined Annotation Dependent Depletion (CADD) scores [6]. Although there is no hard cut-off to determine potential pathogenicity in VUS, a cut-off of 15 for scaled CADD scores has been suggested [6]. Here we report only those variants that have achieved this CADD score cut-off and refer to these as putative (i.e., thought to be but not proven) deleterious variants. Genes with variants specific to CS patients were analysed using the knowledge base of known and predicted protein-protein interactions STRING database (https://string-db.org/). Phenotypes, pathways and cell compartments to which genes carrying putative deleterious (PHRED-scaled CADD scores ≥15) variants belonged were determined using the comprehensive gene set enrichment tool Enrichr (https://maayanlab.cloud/Enrichr/).

## Results

### The study cohort

S2 Table shows clinical data and family history of stroke-related diseases for the 16 CS patients (designated P1 to P16). All except P1 and P16 presented with hypertension; 4 (P3, P11, P12, P13) had diabetes; all had Fazekas scores of 2 or 3 for white matter hyperintensities. All except P10 had a family history of stroke; all except P1, P9 and P10 reported family history of hypertension. Five patients (P2, P3, P4, P7, P11) reported a history of diabetes in a first degree relative; 2 CS patients (P12, P13) had first degree relatives who had suffered heart attacks with myocardial infarctions. Unaffected family members had no comorbities.

### Genetic variants identified

In all, 26 VUS in 19 genes and one asymptomatic heterozygous pathogenic carrier at autosomal recessive *PMM2* were identified in CS patients (Table 1). P2, P5 and P16 had no putative deleterious variants in the targeted panel. Of the 26 VUS, 23 were heterozygous at autosomal genes, one homozygous autosomal variant at *ITGA2B*, and two hemizygous X-linked VUS at *ATP7A* and at *GLA*. Although pathogenic variants at these three genes cause serious monogenic disorders, these CS patients did not present with clinical signs relevant to them. All VUS in CS patients had PHRED-scaled CADD scores ≥15 or were disruptive in-frame deletions and hence have the potential to be deleterious. Nine variants observed in CS patients were also observed in unaffected relatives (Table 2). Given that unaffected relatives were, on average, younger than CS patients we cannot discount the possibility that these variants could contribute to future episodes of CS. We therefore take a conservative approach here in taking forward only the 15 genes with putative deleterious variants specific to the CS group (Table 1), and the 18 genes carrying putative deleterious variants specific to unaffected relatives (Table 2), in gene-set enrichment analysis.

**Table 1:** Pathogenic variants and variants of uncertain significance (VUS) identified in the CS patients.

Genes	Variants	Chromosome Location	Zygosity	ClinVar Classification	CADD Score	Individuals with Variant*
*WFS1*	NM001145853.1: c.2380G > A p.Glu794Lys	chr4: 6303902	Heterozygous (Missense)	VUS	23.0	P1
	**NM_001145853.1:c.2137G > A** **p.Asp713Asn**	chr4: 6303659	Heterozygous (Missense)	VUS	28.0	P8
*NOTCH3*	NM_000435.2: c.3691C > T; p.Arg1231Cys	chr19: 15289863	Heterozygous (Missense)	VUS**	26.9	P3
	**NM_000435.2: c.2824G > T; p.Gly942Cys**	chr19: 15291942	Heterozygous (Missense)	VUS	23.4	P4
	**NM_000435.2: c.6182A > C;** **p.Lys2061Thr**	chr19: 15272257	Heterozygous (Missense)	VUS	24.4	P14
*PKD1*	NM_001009944.2:c.5899G > A p.Val1967Met	chr16: 2159269	Heterozygous (Missense)	VUS	24.6	P10
	NM_001009944.2:c.6439C > T p.Arg2147Trp	chr16: 2158729	Heterozygous (missense)	VUS	NA	P13
	**NM_001009944.2:c.5323G > A** **p.Gly1775Ser**	chr16: 2159845	Heterozygous (missense)	VUS	24.7	P14
*CPS1*	NM_001122633.2:c.2629A > T p.Thr877Ser	chr12: 211481189	Heterozygous (Missense)	VUS	20.6	P3
	NM_001122633.2:c.3331C > T p.Pro1111Ser	chr2: 211512758	Heterozygous (Missense)	VUS	23.4	P3
*DOCK8*	**NM_203447.3: c.4019A > G; p.Tyr1340Cys**	chr9: 420579	Heterozygous (Missense)	VUS	27.3	P9, P15
	NM_203447.3: c.3175C > T; p.Leu1059Phe	chr9: 399200	Heterozygous (missense)	VUS	27.8	P15
*POLG*	NM_001126131.1:c.2978G > A; p.Arg993His	chr15: 89864000	Heterozygous (Missense)	VUS	29.3	P6
*ABCC6*	NM_001171.5: c.1247A > G p.Asp416Gly	chr16: 16291969	Heterozygous (Missense)	VUS	24.7	P6
*YY1*	**NM_003403.4:c.222_224delCCA** **p.His75del**	chr14: 100705787	Heterozygous (Disruptive InDel)	VUS	NA	P7
*F13A1*	NM_000129.3: c.2008G > A p.Gly670Ser	chr6: 6152083	Heterozygous (Missense)	VUS	28.4	P7
*ATP7A*	NM_000052.6: c.1256T > C c.Val419Ala	chrX: 77245374	Hemizygous (Missense)	VUS	23.0	P7
*F5*	NM_000130.4: c.3319G > C p.Asp1107His	chr1: 169511009	Heterozygous (Missense)	VUS	14.9	P9
*JAG1*	NM_000214.2: c.2930A > G p.Glu977Gly	chr20: 10621879	Heterozygous (Missense)	VUS	21.7	P9
*GLA*	NM_000169.2: c.1196G > C; p.Trp399Ser	chrX: 100652891	Hemizygous (Missense)	VUS	19.9	P9
*MTHFR*	**NM_005957.4: c.121C > T;** **p.Arg41Trp**	chr1: 11863053	Heterozygous (Missense)	VUS	26.7	P10
*PMM2*	NM_000303.2: c.338C > T; p.Pro113Leu	chr16: 8900255	Heterozygous (Missense)	Pathogenic (Carrier)	29.2	P11
*PDE6A*	**NM_000440.2:c.363_365delCGA;p.Glu122del**	chr5: 149323871	Heterozygous (Disruptive InDel)	VUS	NA	P11
*ITGA2B*	NM_000419.4: c.1374C > G; p.Ile458Met	chr17: 42458266	Homozygous (Missense)	VUS	19.1	P12
*PON1*	NM_000446.5: c.74 + 5G > A;	chr7: 94953709	Heterozygous (Splice site)	VUS	21.3	P14
*RNF213*	**NM_001256071.2: c.11470C > T;** **p.Arg3824Cys**	chr17: 78337016	Heterozygous (Missense)	VUS	28.6	P14

* P1-P15 refer to patients; ** One report of pathogenicity for this variant in ClinVar, 9 of VUS, 1 of benign. NA = unavailable. Bold indicates variants shared in common with unaffected relative (see S3 Table).

**Table 2:** Pathogenic and variants of uncertain significance (VUS) identified in unaffected relatives.

Genes	Variants*	Chromosome Location	Zygosity	Classification	CADD Score	Individuals with Variant**
*YY1*	**NM_003403.4:c.222_224delCCA;** **p.His75del**	chr14: 100705787	Heterozygous (In-frame Del)	VUS	NA	C4, C7, P7
*NOTCH3*	**NM-000435.2: c.2824G > T;** **p.Gly942Cys**	chr19: 15291942	Heterozygous (Missense)	VUS	23.4	C4, P4
	**NM_000435.2: c.6182A > C;** **p.Lys2061Thr**	chr19: 15272257	Heterozygous (Missense)	VUS	24.4	C16. P14
*PKD1*	NM_001009944.2: c.8593C > T; p.Arg2865Trp	chr16: 2153465	Heterozygous (Missense)	VUS	NA	C15
	**NM_001009944.2:c.5323G > A** **p.Gly1775Ser**	chr16: 2159845	Heterozygous (Missense)	VUS	24.7	C16, P14
*FLNA*	NM_001110556.1:c.4759C > T; c.4759C > T; p. Pro1587Ser	chrX: 153585988	Hemizygous (Missense)	VUS	24.5	C1
*PRKAG2*	NM_016203.3: c.1400G > A; p. Gly467Glu	chr7: 151262469	Heterozygous (Missense)	VUS	34.0	C1
*PDE11A*	NM_016953.3: c.2031G > A; c.2031G > A; p. Ala678Thr	chr2: 178592397	Heterozygous (Missense)	VUS	22.7	C3
*HBB*	NM_00518.4: c.27dupG; p. Ser10fs	chr11: 5248224	Heterozygous (Frameshift)	Pathogenic	NA	C3
*PRKCH*	NM_006255.4: c.56C > T; p. Ala19Val	chr14: 6178875	Heterozygous (Missense))	VUS	NA	C4
*F7*	NM_000131.4: c.830A > G; p.Asp277Gly	chr13: 113772751	Heterozygous (Missense)	VUS	22.1	C5
*HDAC*	NM_006037.3: c.3212delA; p.Lys1071fs	chr2: 239975158	Heterozygous (Frameshift)	VUS	NA	C5
*BMPR2*	NM_001204.6: c.622-2A > G	chr2: 203383543	Heterozygous (Splice Site)	VUS	35	C5
*COL1A1*	NM_000088.3: c.613C > G; p. Pro205Ala	chr17: 48275339	Heterozygous (Missense)	VUS	18.8	C6
*ZSWIM6*	NM_020928.1: c.2150G > A; p. Arg717Gln	chr5: 60827457	Heterozygous (Missense)	VUS	33.0	C7
*WFS1*	**NM_001145853.1:c.2137G > A** **p.Asp713Asn**	chr4: 6303659	Heterozygous (Missense)	VUS	28.0	C8, P8
*PDE3A*	NM_000921.4: c.3371A > G; p. Glu1124Gly	chr12: 20833150	Heterozygous (Missense)	VUS	26.4	C9
*CHD1*	NM_001270.2: c.1045T > A; p. Leu349Met	chr5: 98235224	Heterozygous (Missense)	VUS	22.1	C9
*MTHFR*	**NM_005957.4: c.121C > T;** **p. Arg41Trp**	chr1: 11863053	Heterozygous (Missense)	VUS	26.7	C10, P10
*ENG*	NM_001114753.2: c.1724_1726delTCA; p. Ile575del	chr9: 130579442	Heterozygous (In-frame Del)	VUS	NA	C11
*MYLK*	NM_053025.3: c.3748C > T; p. Arg1250Cys	chr3: 123385249	Heterozygous (Missense)	VUS	6.8	C11
*PDE6A*	**NM_000440.2: c.363_365delCGA;** **p. Glu122del**	chr5: 149323871	Heterozygous (In-frame Del)	VUS	NA	C12, P11
*CACNA1A*	NM_023035.2: c.6857delG; p.Gly2286fs	chr19: 13318802	Heterozygous (Frameshift)	VUS	NA	C13
*KCNA5*	NM_002234.3: c.1208G > T; p.Arg403Leu	chr12: 5154521	Heterozygous (Missense)	VUS	32.0	C15
*DOCK8*	**NM_203447.3: c.4019A > G;** **p.Tyr1340Cys**	chr9: 420579	Heterozygous (Missense)	VUS	27.3	C15, P9, P15
*RNF213*	**NM_001256071.2: c.11470C > T;** **p.Arg3824Cys**	chr17: 78337016	Heterozygous (Missense)	VUS	28.6	C16, P14
*SERPIND1*	NM_000185.3: c.488G > A p.Gly163Asp	chr22: 21134088	Heterozygous (Missense)	VUS	23.6	C16
*COL1A2*	NM_000089.3: c.2005C > T; p.Pro669Ser	chr7: 94047844	Heterozygous (Missense)	VUS	9.8	C16

*Bold indicates variants identified in common with the CS patient group. ** C1-C16 refer to unaffected relatives. P1-P16 refer to patients. NA = not available.

### Gene set enrichment and STRING analyses

**Fig 1:**
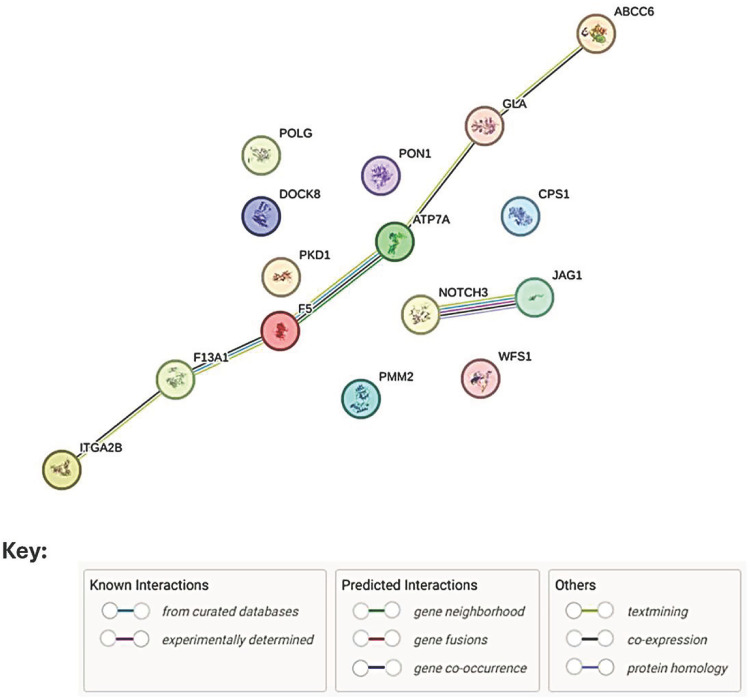
Results of STRING analysis for 15 genes with VUS specific to CS patients. Nodes (= query proteins) are represented by coloured circles; filled nodes indicate that the protein structure is known or predicted. Protein-protein interactions are represented by coloured lines as indicated in the key. These interactions indicated that proteins contribute to a shared function but does not necessarily mean they are physically binding each other (https://string-db.org/).

Putative deleterious variants could contribute to genetic risk either as complex heterozygotes in a single gene (e.g., Table 1 P3 at *CPS1*; P15 at *DOCK8*) or at interacting proteins in common pathways. Gene-set enrichment analysis in Enrichr using the 15 genes carrying putative deleterious variants specific to CS patients (Table 3) showed significant enrichment for gene-sets in phenotypes (e.g., hemorrhage; abnormal blood coagulation; dilated aorta), pathways (e.g., common pathway of fibrin clot formation; platelet degranulation; response to elevated platelet cytosolic Ca^2+^), and cellular components (e.g., platelet alpha granule) that were not observed in Enrichr analysis of 18 genes carrying putative deleterious variants specific to unaffected relatives (S3 Table). This is of interest but should be tempered by the knowledge that our targeted gene panel was selected on the basis of prior association with stroke in other studies. The observations are therefore exploratory and provide a basis for hypothesis generation. STRING analysis demonstrated that 6 of the genes common across the CS patient enriched gene-sets, *ITGA2B*, *F13A1*, *F5, ATP7A*, *GLA* and *ABCC6,* interact at the protein level (Fig 1). This result will again reflect the limitations of our targeted gene study where the gene-set entered into the STRING analysis was constrained by the targeted analysis of the whole exome data. Nevertheless, the STRING analysis provides provisional functional support that patients who carry putative deleterious variants at more than one of these genes, e.g., P7 with variants at *F13A1* and *ATP7A*, could be investigated for a blended deleterious variant phenotype involving these genes. Indeed, multiple patients carried putative deleterious variants across more than one gene in these enriched gene-sets (Table 1).

**Table 3:** EnrichR analysis.

Table and Term	Overlap	P-value	Odds Ratio	Combined Score	Genes
**MGI Mammalian Phenotype Level 4**					
MP:0001914 Hemorrhage	7 of 325	1.69E-09	54.1	1095	*JAG1;PMM2;ITGA2B;F13A1;ATP7A;PKD1;F5*
MP:0002551 Abnormal Blood Coagulation	3 of 51	6.96E-06	103.8	1233	*ITGA2B;F13A1;F5*
MP:0006278 Aortic Aneurysm	2 of 8	1.47E-05	512.3	5701	*ATP7A;PKD1*
MP:0031152 Subcutaneous Hemorrhage	2 of 9	1.88E-05	439.1	4777	*ITGA2B;F13A1*
MP:0005435/5243 Hemoperitoneum/Hemothorax	2 of 10	2.35E-05	384.1	4094	*F13A1;ATP7A*
MP:0004363 Stria Vascularis Degeneration	2 of 13	4.08E-05	279.4	2823	*WFS1;POLG*
MP:0010574 Dilated Aorta	2 of 15	5.48E-05	236.4	2319	*ATP7A;GLA*
MP:0003674 Oxidative Stress	3 of127	1.08E-04	40.0	366	*PON1;ATP7A;POLG*
MP:0002833 Increased Heart Weight	4 of 360	1.20E-04	20.1	181	*NOTCH3;PKD1;GLA;POLG*
**Go Cellular component 2023**					
Platelet Alpha Granule (GO:0031091)	3 of 89	3.73E-05	57.8	590	*ITGA2B;F13A1;F5*
**Jensen Compartments**					
Platelet alpha granule	3 of 72	1.97E-05	72.2	782	*ITGA2B;F13A1;F5*
**Jensen Diseases**					
Cerebrovascular disease	5 of 225	4.72E-07	41.6	704	*NOTCH3;ITGA2B;PON1;GLA;F5*
Cadasil	2 of 9	1.88E-05	439.1	4776	*NOTCH3;JAG1*
**Reactome 2022**					
R-HSA-114608 Platelet Degranulation	3 of128	1.10E-04	39.7	362	*ITGA2B;F13A1;F5*
R-HSA-140875 Common Pathway of Fibrin Clot Formation	2 of 22	1.20E-04	153.6	1386	*F13A1;F5*
R-HSA-76005 Response to Elevated Platelet Cytosolic Ca2+	3 of 133	1.23E-04	38.2	343	*ITGA2B;F13A1;F5*
R-HSA-9013507 NOTCH3 Activation	2 of 25	1.56E-04	133.5	1170	*NOTCH3;JAG1*
R-HSA-140877 Formation of Fibrin Clot (Clotting Cascade)	2 of 39	3.83E-04	82.9	653	*F13A1;F5*
**Human Phenotype Ontology**					
Stroke (HP:0001297)	7 of 33	1.07E-18	672	24698	NOTCH3;JAG1;CPS1;WFS1;ABCC6;PMM2;GLA
Autosomal dominant inheritance (HP:0000006)	10 of 1134	7.64E-10	33.6	704	*NOTCH3;JAG1;WFS1;DOCK8;ABCC6;* *ITGA2B;PON1;PKD1;F5;POLG*
Autosomal recessive inheritance (HP:0000007)	9 of 1722	7.37E-08	14.7	241	*CPS1;WFS1;DOCK8;ABCC6;PMM2;* *ITGA2B;F13A1;F5;POLG*
Aneurysm (HP:0002617)	3 of 39	3.07E-06	138.5	1759	*ABCC6;ATP7A;PKD1*
Myocardial infarction (HP:0001658)	2 of 11	2.88E-05	341.5	3570	*ABCC6;GLA*
Abnormality of the common coagulation pathway (HP:0010990)	2 of 17	7.09E-05	204.8	1957	*F13A1; F5*
Congestive heart failure (HP:0001635)	3 of 128	1.10E-04	39.7	362	*WFS1;ABCC6;GLA*

Results of Enrichr analysis (P < 0.001; adjusted P-values <0.01) for gene sets identified from 15 genes carrying putative deleterious VUS (missense CADD score ≥15 or in-frame deletions) specific to CS patients.

## Discussion

Here we identified 17 putative deleterious variants specific to Indian CS patients in 15 genes previously associated with stroke. The target gene panel comprised genes predominantly identified through common alleles contributing to complex polygenic inheritance of stroke that could include genes influencing a range of comorbidities. The rare putative deleterious variants identified here have the potential to contribute to less complex oligogenic or monogenic inheritance if confirmed as functionally deleterious. A common theme identified amongst the genes carrying the rare variants specific to CS patients was a role in dysmorphic heart/aorta, abnormal blood coagulation, and/or in platelet function and clot formation. Here we focus our discussion on those genes carrying variants that (a) could contribute to monogenic or oligogenic Mendelian inherited disease, and (b) were highlighted (*ITGA2B*, *F13A1*, *F5, ATP7A, GLA, ABCC8*) as potential interacting proteins in our STRING analysis.

Firstly, three patients were either homozygous (P12 at *ITGA2B*) or hemizygous (P7 at *ATP7A*; P9 at *GLA*) for VUS which could be directly disease causing in monogenic disease. *ITGA2B* encodes the alpha subunit of the platelet membrane adhesive protein receptor complex GPIIb/IIIa. Its expression in ischemic stroke has been previously related to elevated platelet cytosolic Ca^2+^ [7]. *ATP7A* encodes a copper-transporting ATPase that predicts cuproptosis, a novel form of programmed cell death in ischemic stroke [8]. *GLA* encodes the lysosomal hydrolase alpha-galactosidase. Previous exome-based analysis identified two pathogenic variants in *GLA* in 172 ischemic stroke patients [9].

Secondly, carriage of putative deleterious alleles across more than one functionally related gene could contribute to oligogenic inheritance of disease risk. For example, previous studies have shown that a blended phenotype of deleterious variants at *NOTCH3* and *RNF213* is associated with temporal pole infarcts in stroke episodes [10]. In our study, CS patient P7 hemizygous at *ATP7A* also carried a putative deleterious variant at *F13A1* encoding coagulation factor XIII, a key differentially expressed gene associated with ischemic stroke [11]. Similarly, patient P9 hemizygous at *GLA* also carried CS-specific putative deleterious variants at *F5. GLA* and *F5* also fall within our STRING of interacting proteins. *F5* encoding coagulation factor V is a novel biomarker in ischemic stroke [12].

Additional CS patients were heterozygous or compound heterozygous for putative deleterious variants at a number of genes that could be dominant for Mendelian inherited disease. Of interest amongst these, P6 carried a potentially deleterious variant at *ABCC6* which belongs to a family of ATP-binding cassette transmembrane transporters. Known pathogenic variants at *ABCC6* are associated with disorders (OMIM *603234) that include arterial calcification and myocardial infarction. In a study of mutations in Mendelian stroke genes in 1,033 early onset stroke patients, clinically relevant VUS were identified at *ABCC6* (n = 53), *RNF213* (n = 59) and *NOTCH3* (n = 15) [13]. Whilst we have focussed here on the specific gene set identified as potentially interactive in our STRING analysis, all the genes carrying variants specific to the CS patient group are worthy of further investigation.

### Limitations and conclusions

We acknowledge that small sample size was a major limitation of our study. Hence our findings should be viewed as exploratory and hypothesis-generating. A further limitation was the targeted analysis of variants in genes previously associated with stroke. A full and unbiased analysis of rare variants across the whole exome, and in a larger sample of patients, will likely identify more variants in genes potentially contributing to CS in our study population. This untargeted approach would also expand the potential for identification of important pathways and protein interactions that could be important in CS. The 17 potentially clinically relevant variants in 15 genes that we identified in 16 CS patients also require secondary validation using an alternative sequencing platform. If validated, further follow-up studies of extended families as well as functional gene-editing laboratory investigations will be required to determine the clinical significance of these variants. Specifically we identify 6 genes (*ITGA2B*, *F13A1*, *F5, ATP7A, GLA, ABCC6*) encoding interacting proteins in functionally relevant pathways that could be prioritised for follow-up. Overall, our results provide a novel contribution to genetic studies of CS in a geographical region and ethnic group not well studied to date.

## Supporting information

S1 Table Panel of 220 stroke-related genes included during targeted analysis of exome sequencing data. (DOCX)

S2 Table Characteristics, clinical data and family history for stroke and stroke-related risk factors for CS patients. (DOCX)

S3 Table Results of Enrichr analysis (P < 0.001; adjusted P-values <0.01) for top gene sets identified from 18 genes carrying 18 putative deleterious VUS (missense CADD score ≥15 or in-frame deletions) unique to the unaffected relatives of CS patients. (DOCX)
