# Supplementary material for: Short report: Targeted analysis of whole exome sequencing data in Indian cryptogenic stroke patients
Source: PLoS One. 2026 Feb 20;21(2):e0326554. doi: 10.1371/journal.pone.0326554 (PMC12923065; doi:10.1371/journal.pone.0326554)
Supplement: S1 Table — (DOCX) [file pone.0326554.s001.docx]

| ABCC6 | JAM3 | USP8 | F7 | PLOD3 | ENG | PREKAG2 | CECR1 | KIF1B | SOST |
| --- | --- | --- | --- | --- | --- | --- | --- | --- | --- |
| ACAD9 | KCNQ1 | USP9X | F8 | PMM2 | EOGT | PRKCH | CFH | KRAS | SPARC |
| ACE | KRIT1 | VHL | FBN1 | PNP | EPOR | PROC | CFHR1 | KRIT1 | STIM1 |
| ACVRL1 | MAX | WFS1 | FGA | POLG | ERF | PTCHD1 | CFHR3 | LMNA | SUOX |
| ADA | MTHFR | XRCC4 | FGB | PRF1 | F10 | RAB27A | CHD1 | MAX | TBC1D24 |
| ADA2 | MUT | XYLT1 | FGFR1 | PRKAG2 | F13A1 | RBM8A | COL1A1 | MMACHC | TBK1 |
| AGT | MYH11 | XYLT2 | FGG | PRKCH | F2 | SAMD9 | COL1A2 | MMUT | TGFB2 |
| ALOX5AP | NOS3 | YY1 | FLNA | PRKG1 | F5 | SAMD9 | COL3A1 | MTHFR | TGBF3 |
| ALPL | NOTCH3 | ZFHX3 | GALK1 | PROC | F7 | SCN1A | COL4A1 | MUT | TGFBR1 |
| APOE | OTC | ZSWIM6 | GDF2 | PROS1 | FCRL1 | SCN5A | COL4A2 | MYBPC3 | TGFBR2 |
| APP | PAI1 | ABCC6 | GJA1 | PRRT2 | FRCL6 | SERPINC1 | COLGALT1 | MYH11 | TLR3 |
| ARHGAP10 | PAX5 | ACAD9 | GLA | PSEN1 | FGFR1 | SH2B3 | CPS1 | MYLK | TMEM127 |
| ARL17A | PCCA | ACE | GSN | PSEN2 | FLNA | SLC19A2 | CPT2 | MYORG | TRAPPC11 |
| ASS1 | PCCB | ACTA2 | GUCY1A1 | RAB27A | FOXF2 | SLC2A1 | CSNK1D | NF1 | TREX1 |
| ATP1A2 | PCNT | ACVRL1 | GUCY1A3 | RASA1 | GALK1 | SLC2A10 | CST3 | NOS3 | TTR |
| ATP1A3 | PDE10A | ADA2 | GYS1 | RBM8A | GLA | SMARCAL1 | CTC1 | NOTCH3 | TUBB2B |
| BANK1 | PDE11A | ALOX5AP | HABP2 | RET | GUCY1A3 | SMCHD1 | CYP27A1 | NPPA | TWNK |
| CACNA1A | PDE3A | ALPK1 | HBB | RHOBTB2 | GYS1 | SMN14 | DOCK8 | OTC | USP18 |
| CD79A | PDE4D | ALPL | HTR1A | RNF213 | HABP2 | SOST | DYRK1B | P2RY12 | VHL |
| CECR1 | PDE6A | AMACR | HTRA1 | SAMD9 | HBB | SPARC | EBF3 | PCCA | WDR62 |
| CFH | PDE6B | APOA1 | ITGA2 | SCN1A | HDAC4 | STIM1 | ELMO2 | PCCB | WFS1 |
| CFHR1 | PDE6C | APP | ITGA2B | SCN5A | HDAC6 | TGFBR3 | ELN | PCNT | XRCC4 |
| CFHR3 | PDE6D | ASS1 | ITGB3 | SERPINC1 | HDAC8 | TMEM127 | ENG | PDCD10 | XYLT1 |
| CHCHD10 | PDE6G | ATP1A2 | ITM2B | SERPIND1 | HDAC9 | TRAPPC11 | ENPP1 | PDE3A | XYLT2 |
| CHD1 | PDE8B | ATP1A3 | IVD | SH2B3 | HTRA1 | TREML2 | EOGT | PDE4D | YY1AP1 |
| COL3A1 | PIK3R5 | ATP5MK | JAG1 | SLC19A2 | IL1RL2 | TREX1 | EPOR | PDGFB | ZSWIM6 |
| COL4A1 | PITX2 | ATP7A | JAK2 | SLC1A3 | IL6 | TSPAN12 | ERF | PDGFRB |  |
| COL4A2 | PMM2 | BMPR2 | JAM3 | SLC2A1 | ILR4 | TSPAN2 | F10 | PEX11B |  |
| CPS1 | PNP | CACNA1A | KCNA5 | SLC2A10 | ITGA2B | TSPAN7 | F13A1 | PGK1 |  |
| CST3 | POLG | CBL | KCNJ2 | SMAD4 | ITGB3 | TTR | F13B | PGM1 |  |
| EBF3 | PON1 | CBS | KCNK18 | SMARCAL1 | IVD | USP18 | F2 | PIK3R5 |  |
| ELMO2 | PRF1 | CCM2 | KCNQ1 | SNX14 | JAK2 | USP27X | F5 | PKD1 |  |

**S1 Table** Panel of genes included during targeted analysis of exome sequencing data
