# Supplementary material for: Short report: Targeted analysis of whole exome sequencing data in Indian cryptogenic stroke patients
Source: PLoS One. 2026 Feb 20;21(2):e0326554. doi: 10.1371/journal.pone.0326554 (PMC12923065; doi:10.1371/journal.pone.0326554)
Supplement: S2 Table — (DOCX) [file pone.0326554.s002.docx]

**S2 Table** Characteristics, clinical data and family history for stroke and stroke-related risk factors for CS patients

| Variables | Patients (N=16) |
| --- | --- |
| Age (mean ± SD) | 63.38 ± 14.43 |
| Hypertensive | 14 |
| Diabetic | 4 |
| Addiction (smoking/tobacco) | 9 |
| Family history:  Stroke  Diabetic  Hypertensive  Myocardial infarction | 15  5  13  2 |
| mRS (mean ± SD) | 6.33 ± 1.9 |
| NIHSS (mean ± SD) | 2.85 ± 1.46 |
| GCS (mean ± SD) | 14.94 ± 0.25 |
| Fazekas:  0  1  2  3 | 0  0  7  9 |

Abbreviations: mRS = Modified Rankin Scale used to measure the degree of disability in stroke patients; NIHSS = NIH Stroke Scale Score quantifies stroke severity; GCS = Glasgow Coma Scale – a score used to assess level of consciousness; Fazekas = Fazekas scale for white matter lesions.
