# Supplementary material for: Short report: Targeted analysis of whole exome sequencing data in Indian cryptogenic stroke patients
Source: PLoS One. 2026 Feb 20;21(2):e0326554. doi: 10.1371/journal.pone.0326554 (PMC12923065; doi:10.1371/journal.pone.0326554)
Supplement: S3 Table — (DOCX) [file pone.0326554.s003.docx]

**S3 Table** Results of Enrichr analysis (P<0.001; adjusted P-values <0.01) for top gene sets identified from 18 genes carrying 18 putative deleterious VUS (missense CADD score ≥15 or in-frame deletions) unique to the unaffected relatives of CS patients.

| **Table and Term** | **Overlap** | **P-value** | **Odds Ratio** | **Combined Score** | **Genes** |
| --- | --- | --- | --- | --- | --- |
| **MGI Mammalian Phenotype Level 4*** |  |  |  |  |  |
| MP:0002169 No Abnormal Phenotype Detected | 10 of 35 | 1.48E-06 | 11.7 | 157 | *COL1A1;F7;BMPR2;COL1A2;PDE3A;CACNA1A; FLNA;CHD1;MYLK;ENG* |
| **Go Cellular component 2023*** |  |  |  |  |  |
| Endoplasmic Reticulum Lumen (GO:0005788) | 4 of 284 | 1.04E-04 | 20.1 | 184 | *COL1A1;F7;COL1A2;SERPIND1* |
| **Jensen Compartments**** |  |  |  |  |  |
| Extracellular space | 8 of 1348 | 9.91E-06 | 11.1 | 128 | *COL1A1;F7;BMPR2;COL1A2;SERPIND1;PRKAG2;HBB;ENG* |
| Fibrillar collagen trimer; Banded collagen fibril | 2 of 9 | 2.74E-05 | 356.7 | 3747 | *COL1A1;COL1A2* |
| Extracellular region part | 11 of 66 | 8.90E-05 | 6.8 | 63 | *COL1A1;F7;PRKCH;BMPR2;COL1A2;SERPIND1;PRKAG2;HBB; FLNA; MYLK;ENG* |
| **Jensen Diseases**** |  |  |  |  |  |
| Cervicitis | 2 of 5 | 7.64E-06 | 832.5 | 9808 | *SERPIND1;FLNA* |
| Bruck syndrome | 2 of 7 | 1.60E-05 | 499.4 | 5514 | *COL1A1;COL1A2* |
| Kidney cancer | 10 of 84 | 2.07E-05 | 8.5 | 91 | *COL1A1;PDE11A;BMPR2;COL1A2;PDE3A;CACNA1A;FLNA; KCNA5; CHD1;MYLK* |
| **Reactome 2022**** |  |  |  |  |  |
| Hemostasis | 8 of 707 | 7.49E-08 | 22.1 | 362 | *COL1A1;F7;PDE11A;PRKCH;COL1A2;SERPIND1;HBB;FLNA* |
| GP1b-IX-V Activation Signalling | 3 of 12 | 1.34E-07 | 443.8 | 7024 | *COL1A1;COL1A2;FLNA* |
| Enhanced Binding of GP1BA Variant to VWF Multimer Collagen | 2 of 7 | 1.60E-05 | 499.4 | 5514 | *COL1A1;COL1A2* |
| **Human Phenotype Ontology**** |  |  |  |  |  |
| Pulmonary hypertension (HP:0002092) | 6 of 80 | 6.04E-11 | 134.5 | 3165 | *COL1A1;BMPR2;COL1A2;HBB;FLNA;ENG* |
|  |  |  |  |  |  |
| Congestive heart failure (HP:0001635) | 5 of 128 | 7.95E-08 | 62.1 | 1015 | *COL1A1;COL1A2;PRKAG2;FLNA;ENG* |
| Thin skin (HP:0000963) | 4 of 56 | 1.67E-07 | 109.5 | 1711 | *COL1A1;PDE11A;COL1A2;FLNA* |
| Tibial bowing (HP:0002982) | 3 of 20 | 6.91E-07 | 234.9 | 3332 | *COL1A1;COL1A2;FLNA* |
| Stroke (HP:0001297) | 3 of 33 | 3.28E-06 | 133.0 | 1680 | *PRKAG2;FLNA;ENG* |

* Only one gene set at P_adj_<0.01. **Not all gene sets at P_adj_<0.01 shown, our purpose being to demonstrate the difference between unaffected relatives and the CS patient gene sets present in main Table 4. Note that Stroke as an enriched gene set in HPO appears at much lower significance than in CS patients (P=1.07E-16 Main Table 4).
